# Two birds with one stone.–Addressing depressive symptoms, emotional tension and worry improves tinnitus-related distress and affective pain perceptions in patients with chronic tinnitus

**DOI:** 10.1371/journal.pone.0246747

**Published:** 2021-03-11

**Authors:** Benjamin Boecking, Matthias Rose, Petra Brueggemann, Birgit Mazurek

**Affiliations:** 1 Charité–Universitaetsmedizin Berlin—Tinnitus Center, Berlin, Germany; 2 Division of Psychosomatic Medicine, Medical Department, Charité–Universitaetsmedizin Berlin, Berlin, Germany; Universiteit Antwerpen, BELGIUM

## Abstract

**Background:**

Psychological factors link the co-occurrence of tinnitus-related distress and pain perceptions in patients with chronic tinnitus.

**Objective:**

This study examines, if treatment-related changes in these factors ameliorate both tinnitus-related distress and pain perceptions in a sample of patients with chronic tinnitus.

**Methods:**

*N* = 1238 patients with chronic tinnitus provided pre- and post-treatment ratings of tinnitus-related distress and affective or sensory pain perceptions alongside measures of depressive symptoms and perceived stress. Treatment comprised an intensive tinnitus-specific multimodal treatment program. Using serial indirect-effects analyses, we examined association patterns between baseline values and change rates of those variables that were found to respond to treatment.

**Results:**

Small effect sizes emerged for changes in tinnitus-related distress, affective (but not sensory) pain perceptions, depressive symptoms, emotional tension and worry. At pre- or post-treatment respectively, baseline values and change rates intercorrelated. Across timepoints, (1) *baseline* tinnitus-related distress and affective pain perceptions were positively associated with *improvements* in tinnitus-related distress, affective pain perceptions and depressive symptoms. (2) *Baseline* depressive symptoms or emotional tension mediated positive associations between *baseline* tinnitus-related distress and *improvement* in affective pain perceptions. (3) *Change* in depressive symptoms mediated the effect of baseline tinnitus-related distress on *change* in affective pain perceptions–partly through associated change in emotional tension or worry. Mood-independent aspects of emotional tension were negatively associated with improvement in affective pain perceptions.

**Conclusions:**

Depressive symptoms, emotional tension and worry emerge as key predictors of treatment response and transdiagnostic treatment targets for alleviating tinnitus-related distress and functionally associated affective pain perceptions.

## Introduction

Both chronic tinnitus and pain are index symptoms of multifactorially influenced syndromes that combine sensory, neurological and psychological components [1–6].

The majority of people who experience tinnitus report no discomfort following symptom onset [7]; however a proportion of people report increased levels of perceived stress [8–10] or low mood [11–13].

Similarly, pain experiences have long been shown to be considerably influenced by cognitive and affective factors [14–17] including perceived stress [18–20], worry [21, 22], and depressive symptoms [23, 24].

Linking these two constructs, Boecking et al. [25] analysed cross-sectional data from a large sample of 1238 patients with chronic tinnitus and reported that [a] a substantive number of patients described notable levels of pain experiences and [b] this co-occurrence was partly explained by common underlying psychological factors including depressive symptoms, emotional tension, worry, and coping attitudes.

Psychological interventions have been shown to be effective in alleviating both tinnitus-related distress [26–29] and pain experiences [30–32]. Similarly, multimodal treatment approaches have shown promising effects for each symptom cluster [33–37].

Among the tinnitus-focused multimodal therapy concepts, our group offered an intensive tinnitus-specific multimodal treatment program between 2011 and 2015. This treatment approach had been previously shown to successfully reduce tinnitus-related distress [38]. However, no study has since investigated [a] if this treatment is effective in a large clinical sample, [b] if beneficial effects on tinnitus-related distress might extend towards pain experiences due to the psychological overlap between the two symptom clusters, and [c] whether any such joint improvements may be attributable to baseline values of or changes in common underlying psychological factors.

The study sample, data collection procedures and measures were previously described in Boecking et al. [25], which reports cross-sectional data from the same sample at baseline. The previous study established psychological factors as common denominators of tinnitus-related distress and pain perceptions. Building on these findings, the present study focuses on treatment-related changes in tinnitus-related distress, pain perceptions and common underlying psychological factors following an intensive tinnitus-specific multimodal treatment program. The study investigates the following research questions: [1] does the brief, intensive tinnitus-specific multimodal treatment program ameliorate tinnitus-related distress, pain experiences, and common underlying psychological factors; [2] is the relationship between *baseline* tinnitus-related distress and *change* in pain perception associated with *baseline* values or [3] *change rates* of psychological factors; and [4] is the relationship between *change* in tinnitus-related distress and pain perception associated with *change* patterns in psychological factors? Based on the previously demonstrated effectiveness of the multimodal treatment program on alleviating tinnitus-related distress [38], the cross-sectional association of tinnitus-related distress and pain perceptions in patients with chronic tinnitus [25], and the reported mediation of this association through shared psychological factors including depressive symptoms and perceived stress [25], we hypothesize positive associations between baseline and change values of tinnitus-related distress and pain perceptions that we expect to be mediated by baseline and/or change values of the previously identified underlying psychological process variables.

## Materials and methods

### Participants

Data were drawn from a large dataset obtained during routine clinical practice of *N* = 3851 patients with chronic tinnitus who [a] self-referred to the Tinnitus Center at Charité Universitaetsmedizin Berlin between January 2011 and October 2015, [b] suffered from chronic tinnitus (lasting for > 3 months), and [c] were 18 years of age or older. Patients with acute psychotic illness or addiction and insufficient knowledge of the German language were not included in the sample. Given the present paper’s focus on treatment-responses of psychological factors and associated changes in tinnitus-related distress and pain perceptions, patients who did not complete the Tinnitus Questionnaire and the Pain Perception Scale at baseline were excluded (*n* = 2613). The final sample included *N* = 1238 patients (50.4% female), *n =* 1098 and 1039 of whom provided post treatment data for the Tinnitus Questionnaire and Pain Perception Scale respectively (*M*_*included*_ = 50.17; *SD*_*included*_ = 12.02). Excluded cases (*n* = 2613) were slightly, but significantly older than those included in the analysis sample (*M*_*exluded*_ = 51.22; *SD*_*exluded*_ = 13.49; *t*(3849) = -2.34, *p* = .02; see also [25].

### Procedure

Upon arrival at the Tinnitus Center for the start of the treatment program, patients completed a routine questionnaire assessment battery on Acer Pocket PC n300 electronic handheld information devices (cf. [25]). The same measures were completed post treatment. Charité Universitaetsmedizin Berlin’s Ethics Committee granted ethical approval (No: EA1/040/08).

### Treatment

Prior to starting treatment, ear-nose-throat and psychosomatic specialists conducted individual otological, audiological and psychosomatic diagnostics alongside physical examinations. Treatment then comprised an intensive tinnitus-specific multimodal treatment program that included psychoeducation-, cognitive-behaviour therapy-oriented-, relaxation-, audiological- and physiotherapeutic treatment components. Upon beginning treatment, patients were familiarized with progressive muscle relaxation (PMR) by Edmund Jacobson [39] and practiced this relaxation strategy daily throughout therapy. Additionally, patients participated in daily group physiotherapy exercises and were offered two single sessions of physiotherapy each. The psychological aspects of the treatment program included [1] psychoeducation about basic hearing physiology, and the anatomy and function of the auditory system as well as models of stress and stress management, [2] daily auditory training, which comprised audiological defocusing exercises, [3] daily cognitive-behavioral group therapy focusing on dysfunctional cognitions concerning tinnitus, anxieties, sleep disturbances and stress; and [4] two individual psychological consultations that focused on individual difficulties reported by the patients. An interdisciplinary team of trained clinical psychologists or physiotherapists delivered all interventions. Medical professionals were available to address medical issues where applicable.

### Measures

#### Tinnitus-related distress

The German version of the tinnitus questionnaire [TQ; 40] was administered to assess the psychosocial impact of tinnitus symptomatology. It consists of 52 statements that are answered on a 3-point scale (0 = *not true*, 1 = *partly true*, 2 = *true*). The total score uses 40 items—two of them twice—thus yielding a score between zero and 84. The scale’s test-retest reliability is good (total score: *r* = 0.94; [41]), and the TQ has been found to be sensitive to change [42]. In the current sample, the scale’s internal consistency was excellent (α = 0.92).

#### Pain perception

The Pain Perception Scale *(“Schmerzempfindungsskala"-SES;* [43]) measures subjective pain perceptions across an affective and sensory subscale. The former uses 14 items to obtain indications of subjective pain-related affective distress [e.g. “I experience my pain as intolerable”]. The latter uses 10 items to obtain indications of subjective experiences of physically experienced pain sensations [e.g. “I experience my pain as throbbing”]. All items are answered on a 4-point-scale (1 = *does not apply*, 2 = *hardly applies*, 3 = *somewhat applies*, 4 = *completely applies*) with scores ranging from 14–56 [affective pain perception] and 10–40 respectively [sensory pain perception]. The scale’s test-retest reliability is good (*r* = 0.89–0.96) with internal consistency being moderate to high (α = 0.72–0.92; [43]). In the current sample, internal consistencies were excellent (α_affective_ = 0.96; α_sensory_ = 0.90).

#### Psychological comorbidities

Psychological “comorbidities” (i.e. psychological epiphenomena reciprocally associated with chronic tinnitus as the index symptom in focus) were measured using the ICD-10 Symptom Rating [ISR; 44, 45]. The ISR consists of 29 items that are answered on a 5-point-scale (0 = *does not apply*, 1 = *hardly applies*, 2 = *somewhat applies*, 3 = *considerably applies*, 4 = *completely applies*). The measure includes five subscales that measure the presence of depressive, anxiety-related, obsessive, somatoform [including health-anxiety] and eating-related symptoms that are linked to syndromatic diagnostic categories as defined by the International Classification of Diseases-10 [46]. An additional supplementary scale measures various aspects of psychological distress or clinical relevance. Indexing the extent of overall emotional impairment, a total score is calculated that weighs the supplementary scale twice. All indices range from zero to 4. Test-retest reliability is good (*r* = 0.84–0.84; [44]) and the scale has been shown to be sensitive to change [44, 47]. In the current sample, internal consistency was excellent (α = 0.93).

#### Depressive symptoms

Depressive symptoms were measured using the German version of the Center for Epidemiological Studies Depression Scale (“Allgemeine Depressionsskala”-ADS; [48, 49]). The scale features 20 items that measure emotional, motivational, cognitive, somatic and motoric symptoms of low mood on a 4-point-Likert scale (0 = *rarely*, 1 = *sometimes*, 2 = *often*, 3 = *almost always*) yielding a range from zero to 60. Test-retest reliability is moderate (*r* = 0.51–0.67) with internal consistency ranging from 0.85 to 0.92 [49]. In the current sample, internal consistency was acceptable (α = 0.73).

#### Perceived stress

Perceived stress was measured using the German version of the Perceived Stress Questionnaire [PSQ; 50, 51]. The scale contains four dimensions three of which focus on internal stress reactions (tension, worry, [lack of] joy) and one on perceived external stressors (demands). *Tension* explores tense disquietude, exhaustion and lack of relaxation. *Worry* assesses anxious concern for the future, and feelings of desperation and frustration; *joy* assesses positive feelings of challenge, joy, energy, and security and *demands* assesses perceived environmental demands such as lack of time, pressure, and overload. The scale consists of 30 items that are rated on a 4-point scale (1 = *almost never*, 2 = *sometimes*, 3 = *often*, 4 = *almost always*). All indices are linearly transformed to range from 0 to 1 and are subsumed in a total score for which *joy* is recoded. The PSQ has been found to be sensitive to change [50]. In the current sample, internal consistency was excellent (α = 0.90).

#### Coping attitudes

The *Self-Efficacy-Optimism-Pessimism-Scale (“Selbstwirksamkeits-Optimismus-Pessimismus-Skala”-SWOP;* [52]) measures adaptive and maladaptive coping attitudes. The scale comprises nine items that are answered on a 4-point scale (1 = *does not apply*, 2 = *hardly applies*, 3 = *somewhat applies*, 4 = *completely applies*) and that load on three independent scales each ranging between one and 4: self-efficacy, optimism and pessimism. In the current sample, internal consistencies were good, acceptable and questionable respectively (α_self-efficacy_ = 0.82; α_optimism_ = 0.79; α_pessimism_ = 0.65).

Whilst some of the measured questionnaires feature published cut-off scores, we conceptualize all psychological traits as dimensionally distributed along an individual differences continuum [53–56].

### Data analyses

All analyses were conducted using IBM SPSS Statistics for Windows, Version 24. Correlation coefficients between baseline and change values were calculated using Spearman’s *ρ*. Pre- and post-treatment scores were compared using paired-samples *t*-tests. Effect sizes *g* were calculated separately [57]. Estimates were defined as small (0.10<*g*< 0.20), medium (0.21<*g*< 0.30), large (0.31<*g*< 0.40) or very large (*g*> 0.40; [58]).

**Fig 1:**
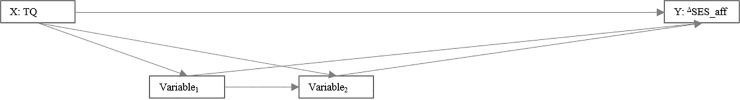
Indirect-effects models. TQ = tinnitus-related distress [Tinnitus Questionnaire (German version)—total score]; SES_aff = affective pain perception [Pain Perception Scale–subscale score]; X = independent variable; Y = dependent variable; Variables 1 and 2: alternate pairs of treatment-responsive process variables (depressivity [Center for Epidemiological Studies Depression Scale–total score or ICD-10 Symptom Rating–subscale score], emotional tension and worry [Perceived Stress Questionnaire–subscale scores]); t_0_ = pre treatment; t_1_ = post treatment; ^Δ^ = change score (t_1_ minus t_0_). Fig 1 illustrates the model specification that investigates if the relationship between *baseline* tinnitus-related distress and *change* in affective pain perception is associated with *baseline* values of psychological process variables.

**Fig 2:**
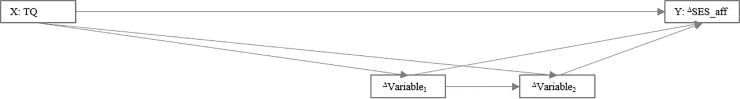
Model specification that investigates if the relationship between baseline tinnitus-related distress and change in affective pain perception is associated with *change* patterns in psychological process variables.

**Fig 3:**
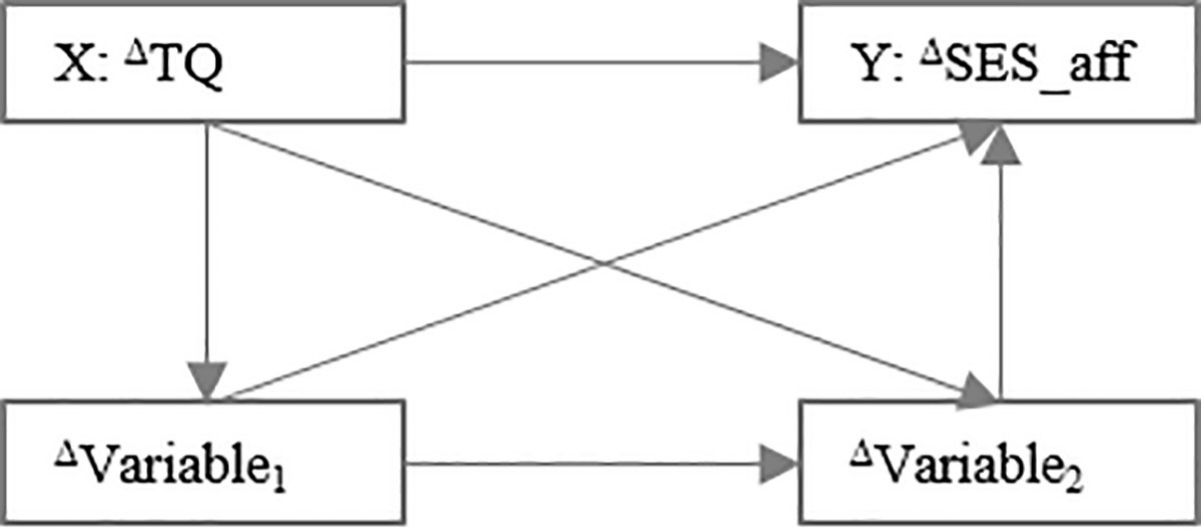
Model specification that investigates if the relationship between *change* in tinnitus-related distress and affective pain perception is associated with *change* patterns in psychological process variables.

The present paper focuses on interactions of treatment-responsive variables in potentially influencing tinnitus-related distress and functionally associated pain perceptions. Consequently, we included only those variables in the indirect-effects analyses that showed treatment-related change with at least small effect sizes. Because sensory pain perception (SES), anxiety-related, obsessive, somatoform and eating-related symptoms (ISR), joy, demands (PSQ), and coping attitudes (SWOP) did not meet this criterion, these variables were dropped from further analyses. Indirect-effects models were computed using Hayes et al.’s *process* macro [59, 60]. These models aimed to examine interactions between baseline values and change rates of tinnitus-related distress, affective pain experiences, and the identified psychological factors. We do explicitly not postulate causality between the independent, mediating and dependent variables [61]. Models were specified within a *main effect* x *paired treatment-responsive factors* for [a] *baseline values* and [b] *change rates*—matrix. Each model featured baseline- or change in tinnitus-related distress or affective pain perception as independent or dependent variables respectively (see Figs 1–3):

## Results

### Descriptive indices

Table 1 provides means and standard deviations for the sample at pre- and post-treatment alongside effect size indicators of change. Small effect sizes *g* emerged for changes in tinnitus-related distress, affective pain perceptions, depressive symptoms, emotional tension and worry.

**Table 1:** Means, standard deviations and effect sizes of change for the sample at pre and post treatment (*N* = 1238 patients with chronic tinnitus).

	Pre		Post		Group effect	Effect size
	*M*	*SD*	*M*	*SD*		*g CI*
**TQ**						
*Total*	*39*.*66*	*16*.*98*	*32*.*63*	*17*.*24*	*F(1*, *1097) = 576*.05^***^	*0*.*37–0*.*45*
**SES**						
*Affective*	*24*.*18*	*10*.*00*	*22*.*69*	*9*.*67*	*F(1*, *1038) = 45*.65^***^	*0*.*11–0*.*20*
Sensory	13.71	4.98	13.59	5.07	F(1, 1038) = 0.89	-0.02–0.07
**ISR**						
Total	.81	.56	.76	.59	F(1, 971) = 19.40^***^	0.05–0.13
*Depressive syndrome*	*1*.*18*	.*91*	*1*.*03*	.*92*	*F(1*, *971) = 58*.14^***^	*0*.*12–0*.*21*
Anxiety-related syndrome	.94	.90	.89	.91	F(1, 971) = 6.71^*^	0.01–0.10
Obsessive-compulsive syndrome	.79	.86	.80	.84	F(1, 971) = 0.55	-0.07–0.04
Somatoform syndrome	.61	.79	.59	.78	F(1, 971) = 1.08	-0.02–0.07
Eating-related syndrome	.68	.80	.64	.82	F(1, 971) = 5.55^*^	0.01–0.09
Supplementary scale	.75	.54	.68	.57	F(1, 971) = 30.93^***^	0.09–0.17
**ADS**						
*Total*	*18*.*23*	*11*.*82*	*13*.*26*	*10*.*83*	*F(1*, *1009) = 349*.32^***^	*0*.*39–0*.*49*
**PSQ**						
Total	.46	.18	.43	.19	F(1, 1097) = 121.40^***^	0.13–0.19
*Tension*	.*59*	.*22*	.*53*	.*23*	*F(1*, *1097) = 163*.68^***^	*0*.*23–0*.*31*
*Worry*	.*40*	.*22*	.*36*	.*23*	*F(1*, *1097) = 69*.17^***^	*0*.*14–0*.*21*
Joy	.48	.22	.50	.24	F(1, 1097) = 34.95^***^	0.05–0.12
Demands	.50	.23	.47	.22	F(1, 1097) = 49.18^***^	0.09–0.17
**SWOP**						
Self-efficacy	2.76	.57	2.83	.58	F(1, 1087) = 37.20^***^	0.08–0.16
Optimism	2.72	.75	2.80	.76	F(1, 1087) = 33.15^***^	0.06–0.15
Pessimism	2.12	.71	2.15	.73	F(1, 1087) = 1.71	-0.09-[-0.01]

*Notes*: *M* = mean; *SD* = standard deviation; *CI* = 95% Confidence Interval; *g* = Hedge’s *g*; TQ = Tinnitus Questionnaire–German version (tinnitus-related distress); SES = Pain Perception Scale (pain perception), ISR = ICD-10 Symptom Rating (psychological ‘comorbidities’), ADS = Center for Epidemiological Studies Depression Scale (depressive symptoms), PSQ = Perceived Stress Questionnaire (perceived stress), SWOP = Self-Efficacy-Optimism-Pessimism-Scale (coping attitudes). Variables that differ on the *p* < .001 level and with an at least small effect size (defined as confidence intervals of *g* lying between 0.10 and 0.20 [58]) are *italicized*.

^*^ = *p* < .05

^***^ = *p* < .001

### Indirect-effects analyses

Most baseline values and change rates intercorrelated except for change in emotional tension, which appeared to ensue irrespective of most measured variables’ baseline values (Table 2).

**Table 2:** Correlation coefficients [*ρ*] for baseline values and change rates of those variables that responded to treatment.

	t_0_					^Δ^ [t_1_ –t_0;_ negative values indicate improvement]					
	SES_aff	ADS	ISR_D	PSQ_T	PSQ_W	^Δ^TQ_total	^Δ^SES_aff	^Δ^ADS	^Δ^ISR_D	^Δ^PSQ_T	^Δ^PSQ_W
TQ_total	.53***	.64***	.55***	.52***	.50***	-.25***	-.09**	-.20***			
SES_aff		.50***	.49***	.42***	.41***	-.09**	-.41***	.-21***	-.08*		
ADS			.81***	.69***	.73***	-.08**	-.08**	-.48***	-.12***		
ISR-D				.67***	.71***	-.11***	-.12***	-.35***	-.34***		-.06*
PSQ-T					.67***	-.12***	-.10**	-.32***	-.11***	-.28***	-.09**
PSQ-W						-.10**		-.28***	-.09*		-.26***
^Δ^TQ_total							.26***	.35***	.32***	.36***	.35***
^Δ^SES_aff								.30***	.29***	.22***	.26***
^Δ^ADS									.38***	.29***	.23***
^Δ^ISR-D										.27***	.23***
^Δ^PSQ-T											.39***

*Notes*. Only significant effects are featured.

*** = *p* < .001

** = *p* < .01

* = *p* < .05.

TQ_total = tinnitus-related distress [Tinnitus Questionnaire (German version)—total score]; SES_aff = affective pain perception [Pain Perception Scale–subscale score]; ADS = depressive symptoms [“Allgemeine Depressionsskala”–total score]; ISR-D = depressive symptoms [ICD-10 Symptom Rating–subscale score]; PSQ-T = emotional tension; PSQ-W = worry [Perceived Stress Questionnaire—subscale scores]); t_0_ = pre-treatment; t_1_ = post-treatment; ^Δ^ = change score (t_1_ minus t_0_).

**Fig 4:**
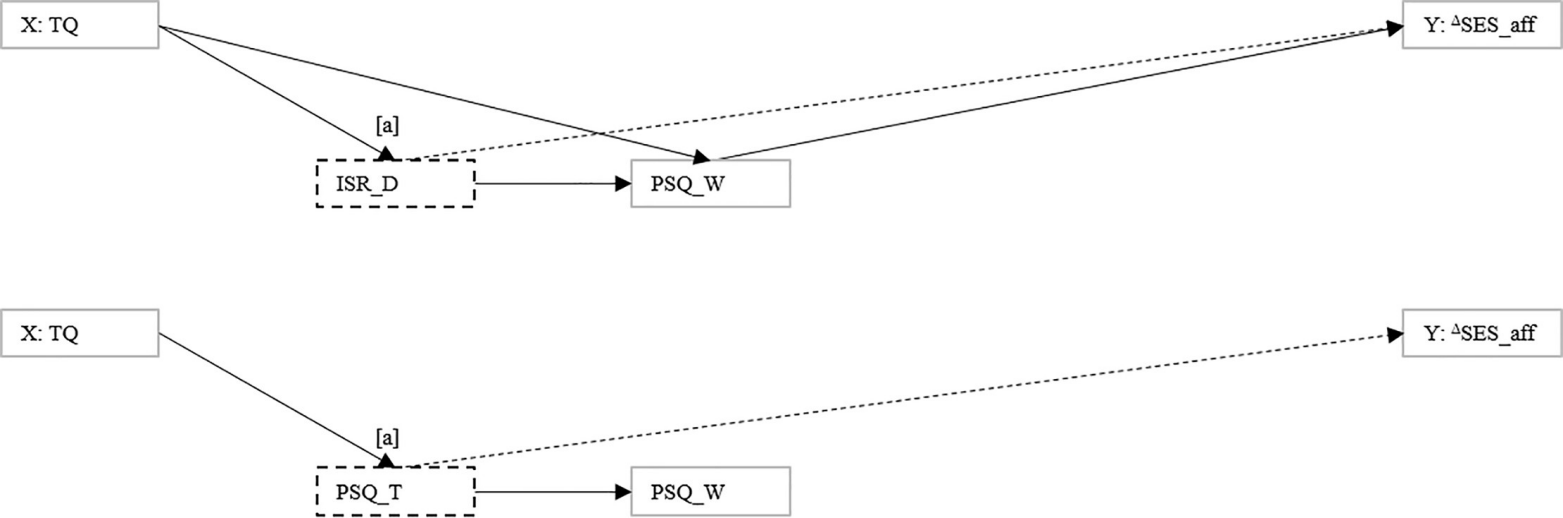
Indirect-effects models. Black continuous arrows indicate significant positive; black dotted arrows significant negative effects. Black continuous box frames indicate significant positive; black dotted box frames significant negative indirect effects through the respective variables. TQ = tinnitus-related distress; SES_aff = affective pain perception; ADS-L and ISR_D = depressivity; PSQ_T = emotional tension; PSQ_W = worry; X = independent variable; Y = dependent variable; t_0_ = pre treatment; t_1_ = post treatment; ^Δ^ = change score (t_1_ minus t_0_; negative values denote improvement). For those models that yielded significant indirect effects, all significant effects are illustrated. Fig 4 shows *the relationship between baseline tinnitus-related distress and change in affective pain perception as influenced by baseline values of psychological process variables*.

**Fig 5:**
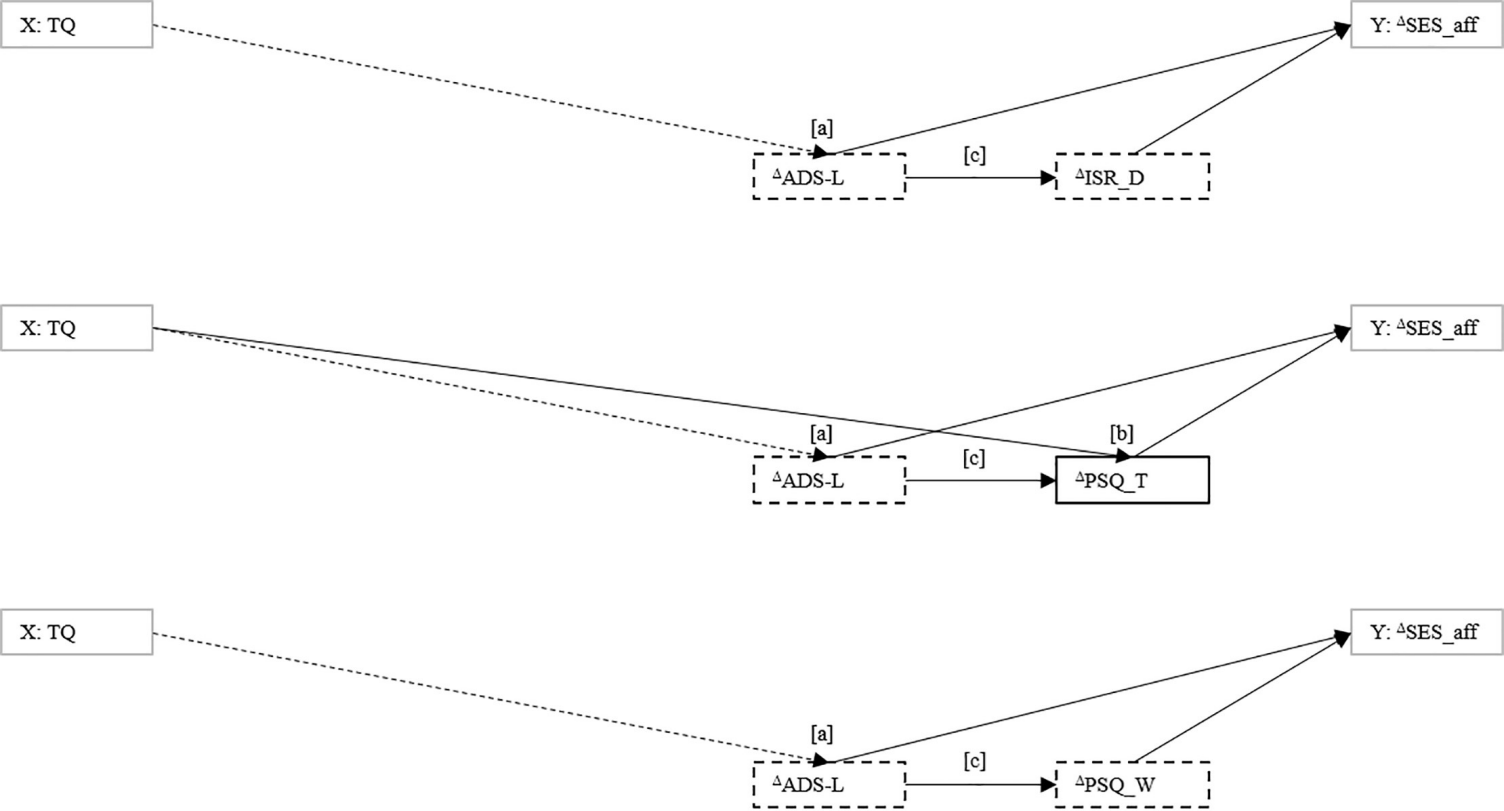
The relationship between baseline tinnitus-related distress and change in affective pain perception as mediated by change patterns in psychological process variables. The positive relationship between baseline tinnitus-related distress and improvement in affective pain perception was mediated by pathways involving [a] a positive association between baseline tinnitus-related distress and improvement in depressivity (ADS-L) or [b] a negative association between baseline tinnitus-related distress and improvement in emotional tension that were both positively associated with change in affective pain perception. Significant three-way interactions further revealed pathways involving positive associations between baseline tinnitus-related distress and [c] improvements in depressivity (ADS-L), *additional* changes in depressivity (ISR_D), emotional tension or worry, and change in affective pain perception.

**Fig 6:**
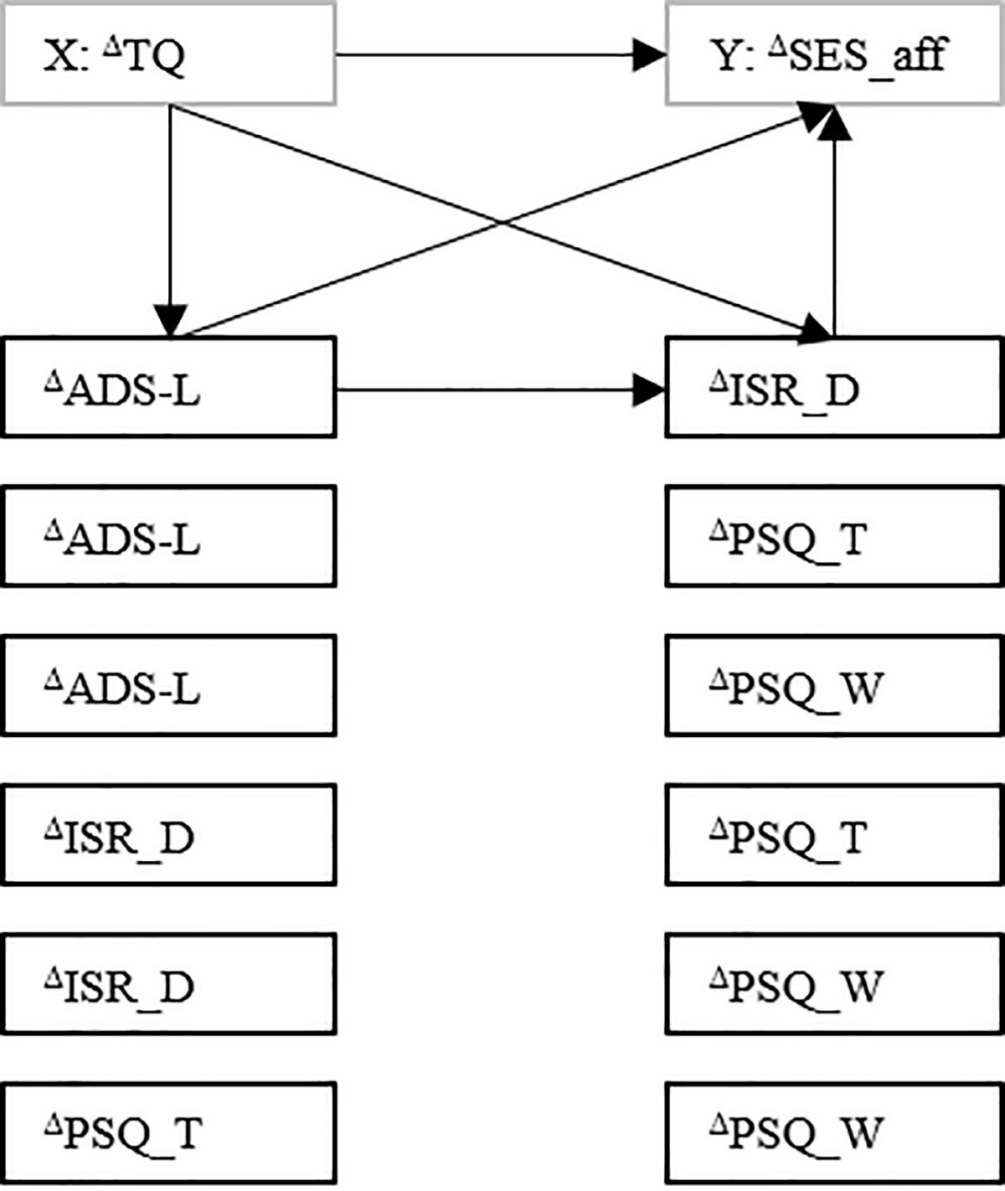
The relationship between change in tinnitus-related distress and affective pain perception as influenced by change patterns in psychological process variables. The positive relationship between changes in tinnitus-related distress and affective pain perception was positively associated with (relationships between) contemporaneous changes in all process variables.

Figs 4–6 outline significant effects for the indirect-effects models that were specified within a *main effect* [TQ−^Δ^SES_aff, ^Δ^TQ−^Δ^SES_aff] x *paired treatment-responsive factors* [ADS–ISR-D, ADS–PSQ-T, ADS–PSQ-W, ISR-D–PSQ-T, ISR-D–PSQ-W, PSQ-T–PSQ-W] for [a] *baseline values* and [b] *change rates*—matrix. Path coefficients are given in the S1 Table.

The positive relationship between baseline tinnitus-related distress and improvement in affective pain perception was mediated by pathways involving positive associations between baseline tinnitus-related distress and [a] baseline depressivity (ISR_D) or emotional tension each of which were positively associated with improvement in affective pain perception. Moreover, controlling for baseline tinnitus-related distress and depressivity, baseline worry was negatively associated with improvement in affective pain perception.

## Discussion

We previously reported that psychological factors—notably depressive symptoms, emotional tension and worry—underlay an association of tinnitus-related distress and affective as well as sensory pain perceptions in patients with chronic tinnitus [25]. Building on these findings, the present study investigated if an intensive tinnitus-specific multimodal treatment program [a] alleviated tinnitus-related distress, [b] alongside conceptually and functionally similar pain perceptions; and [c] may have exerted such a joint effect through addressing common underlying psychological factors.

Does the brief, intensive tinnitus-specific multimodal treatment program ameliorate tinnitus-related distress, pain experiences, and common underlying psychological factors?

The multimodal treatment program—which included psychosomatic diagnostics, psychoeducation components, cognitive-behaviour therapy-oriented interventions, relaxation exercises, and physiotherapy—was associated with at least small-effect sized improvements in tinnitus-related distress, affective (but not sensory) pain perceptions, depressive symptoms, emotional tension and worry. This finding is in keeping with previous research highlighting the rather psychological makeup of affective—relative to sensory—pain perception, which is consequentially more likely to respond to psychologically focused interventions like the one examined in this paper [62–65]. Similarly, a recent study reported that subgroups of patients with varying levels of affective pain experiences reported analogous levels of depressive difficulties. For sensory pain experiences, by contrast, respective mappings appeared more heterogeneous [66]. No changes in coping attitudes were observed. Whilst optimism [67, 68] or lowered self-efficacy beliefs [25] have been shown to contribute to tinnitus-related distress or chronic illness more generally [69, 70], the treatment program did not have beneficial effects on these constructs–possibly owed to its short duration. Whilst addressing these constructs within a broader psychotherapeutic context may yield additional effects [71–73] the scales’ low internal consistencies further suggests that alternative measures may be more suitable when wishing to test related hypotheses.

Both baseline values and change rates were positively intercorrelated suggesting a conceptual overlap between the measured constructs–or possibly a general factor of psychopathology that may reflect common aspects of tinnitus-related distress, depressive symptoms, worry, affective pain experiences and emotional tension on an individual differences continuum [74–76]. The treatment program may facilitate well-being and improve tinnitus-related distress by targeting [a] shared variance of the measured constructs, or [b] specific variance of each measured factor—an explanation that seems somewhat less likely given that the psychological interventions were more transdiagnostic in nature.

At baseline, most patients showed only mild-to-moderate symptom severity levels, thus restricting the potential of therapeutic improvement and raising the possibility of Type-II errors for some of the measured indices. Notwithstanding, symptom severity was positively associated with improvement in the measured key indices. Multimodal treatment as examined in the present paper thus appears to benefit patients along the presenting range of symptom severity. Interestingly, treatment-related improvements in emotional tension and—to a lesser extent—worry appeared to occur somewhat irrespective of the measured constructs’ baseline values. These factors thus emerge as potential common “third variables” whose therapeutic targeting may bear beneficial effects on emotional distress as reflected in the measured variables. Supporting this view, emotional exhaustion–a component of emotional tension as measured in the present study–has been previously found to be a common risk factor for burnout and posttraumatic stress disorder following triggering circumstances [77], hyperacusis in women following triggering circumstances [78] and chronic pain [79, 80]. Similarly, worry has been identified as a transdiagnostic construct common to depressive symptoms, anxiety [81, 82] and pain [3] possibly serving a psychological function to reduce negative affect associated with underlying interpretational thought-memory-interactions [83].

Is the relationship between baseline tinnitus-related distress and change in pain perception associated with baseline values of psychological factors?

The association between baseline tinnitus-related distress and change in affective pain perception was mediated by baseline levels of depressive symptoms or emotional tension. This finding is in keeping with previous reports whereby depressive symptoms accounted for considerable shared variance in both tinnitus-related distress, pain experiences and psychological epiphenomena–sometimes somewhat misleadingly labelled comorbidities (i.e. suggesting separate or separable illness entities; [25]). When controlling for baseline tinnitus-related distress and depressive symptoms, worry was negatively associated with improvements in affective pain perception. Worry has long been conceptualized as part of a cognitive-attentional syndrome that maintains emotional distress [84, 85]–possibly by functioning to avoid the emotional processing of underlying distressing experiences [86, 87]. Studies from older adult populations have further identified worry as a predictor of treatment outcome [88, 89]. Worry therefore presents as an important treatment target–alongside depressive symptoms–whose modification is likely to benefit patients across different somatization phenomena. Psychologically anchored treatment approaches may thus benefit from formulating and addressing interactions of negative affect, emotional avoidance / worry and depressive symptoms across the spectra of treatment programs for patients with chronic tinnitus [e.g. 90].

Is the relationship between baseline tinnitus-related distress and change in pain perception associated with change rates of psychological factors?

Baseline tinnitus-related distress was positively associated with improvement in affective pain experiences alongside *more* improvement in depressive symptoms—as measured by two different questionnaires. Whilst the treatment program may have addressed parts of the common or specific variance of each factor (see above), the used measures of depressive symptoms further appear to reflect different aspects of low mood [91] that bear conjoint importance for tinnitus-related distress. Indeed, whilst the ICD-10 Symptom Rating preliminary uses mood-related items (e.g. “During the last week, my mood was low and depressed”), the ADS further incorporates items inquiring about somatic (e.g. “During the last week, I hardly had any appetite”) and intra-interpersonal expressions of depressive symptoms (e.g. “During the last week, other people were unfriendly to me”). Patients’ experiences of depressed mood seem to incorporate symptoms across all of these dimensions with some patients possibly endorsing more somatic-, and others more cognitive-emotional conceptualizations. In addition, patients’ pain symptomatology–although partly influenced by depressive symptoms as influencing factor–may conversely confound depressive symptoms ratings by influencing patients’ response patterns on the applied questionnaires (for discussions of this difficulty in other areas, see e.g. [92–94]).

Baseline tinnitus-related distress was further associated with improvement in affective pain experiences alongside *less* improvement in emotional tension. Thus, the specific variance of emotional tension (as a dimension of “perceived stress”) emerges as important when conceptualizing and addressing tinnitus-related distress and pain symptomatology. Importantly, any such “perceived stress” conceptualization ought to occur within psychological frameworks that consider psychological vulnerability-stress interactions [95], personality dimensions [96], and individual constructions of meaning; *not* seemingly “external” factors such as “workload” [97, 98].

Is the relationship between change in tinnitus-related distress and pain perception associated with change patterns in psychological factors?

Finally, the relationship between change in tinnitus-related distress and affective pain perception was associated with change patterns in all psychological factors. The observed interdependencies of change again challenge the notion and helpfulness of a “disease entity” approach wherein “tinnitus” and “comorbidities” are conceptualized as interdependently connected, yet separate illness entities. Rather, dimensional, empirically defined conceptualizations of mental health difficulties [55, 99–101] are more suitable in understanding the interplay of factors that may underlie both symptom maintenance and change as observed in the present study.

Transdiagnostic approaches (that accommodate specific factors that are characteristic of certain medical conditions, but focus on transdiagnostic psychological mechanisms) have the potential to alleviate distress across a range of functionally associated, somatoform symptom clusters; particularly if overlap exists in cognitive-emotional or behavioural distress expressions or responses [102]. Importantly, psychological interventions that aim to address emotional tension or worry ought to facilitate a process of “meaning-making” [103–106]. Herein, biographical perspectives are considered alongside current life stressors and cognitive-emotional as well as behavioural coping attempts in order to understand the functions of and “chip away” at the maintaining factors of persistent negative affect.

Limitations of the present study include the absence of a control group as well as its two-timepoint design: the observed changes are confounded by the passage of time, spontaneous recovery, non-specific effects or other unknown factors. Thus, the specific efficacy of the examined treatment program remains to be demonstrated. Similarly, the estimated indirect effects do not imply “true mediation” and cannot be interpreted in a causal manner—as change in the independent variable should temporally precede change in the mediator which should precede change in the outcome variable [61]—a postulate that can only be examined within prospective studies featuring multi-timepoint measurements. Overall, observed effect sizes were small. Given the overall mild–to–moderate baseline symptom severity—and associated variance limitations—results need to be interpreted with caution. Notwithstanding, the present study is the first to demonstrate a joint tinnitus and pain-related benefit in a psychologically anchored, multimodal treatment program. Refocusing treatment efforts on transdiagnostic cognitive-emotional factors may thus benefit a variety of ‘co-morbid’ patient populations.

## Conclusions

The present study highlights the roles of depressive symptoms, emotional tension and worry as both predictors of treatment outcome and transdiagnostic treatment targets in a patient population with chronic tinnitus, tinnitus-related distress and co-occurring affective pain experiences. Idiosyncratic interactions of these factors should be [a] empirically conceptualized using dimensional frameworks of psychopathology [55, 100, 101] rather than categorical psychiatric illness-models and [b] clinically addressed within meaning-making, transdiagnostic psychological treatment frameworks that allow for the formulation and treatment of cognitive-emotional and behavioural expressions of individual vulnerability-stress interactions [107–109].

## Supporting information

S1 Table Path coefficients for significant indirect effects. (DOCX)
