# Supplementary material for: Two birds with one stone.–Addressing depressive symptoms, emotional tension and worry improves tinnitus-related distress and affective pain perceptions in patients with chronic tinnitus
Source: PLoS One. 2021 Mar 11;16(3):e0246747. doi: 10.1371/journal.pone.0246747 (PMC7951911; doi:10.1371/journal.pone.0246747)
Supplement: S1 Table — (DOCX) [file pone.0246747.s001.docx]

**S1 Table.** Path coefficients for significant effects.

*Panel a: Independent variable: TQ; first intermediary variable: baseline process variable_1_; second intermediary variable: baseline process variable_2_; dependent variable:* ^Δ^*SES_aff.*

|  |  | *a_1_* | *se* | *LLCI* | *ULCI* | *a_2_* | *se* | *LLCI* | *ULCI* | *a_3_* | *se* | *LLCI* | *ULCI* | *b_1_* | *se* | *LLCI* | *ULCI* | *b_2_* | *se* | *LLCI* | *ULCI* |
| --- | --- | --- | --- | --- | --- | --- | --- | --- | --- | --- | --- | --- | --- | --- | --- | --- | --- | --- | --- | --- | --- |
| **ISR-D** | **PSQ-W** | *.030* | *.001* | *.027* | *.033* | *.002* | *.000* | *.001* | *.003* | *.155* | *.007* | *.143* | *.168* | *-1.259* | *.368* | *-1.980* | *-.537* | *2.887* | *1.433* | *.074* | *5.699* |
| **PSQ-T** | **PSQ-W** | *.007* | *.000* | *.006* | *.008* | *.003* | *.000* | *.002* | *.004* | *.550* | *.027* | *.498* | *.602* | *-3.647* | *1.374* | *-6.343* | *-.950* |  |  |  |  |

| **Indirect effects** | | *a_1_ b_1_* | *se* | *LLCI* | *ULCI* | *a_2_ b_2_* | *se* | *LLCI* | *ULCI* | *a_1_ a_3_ b_2_* | *se* | *LLCI* | *ULCI* |
| --- | --- | --- | --- | --- | --- | --- | --- | --- | --- | --- | --- | --- | --- |
| **ISR-D** | **PSQ-W** | *-.037* | *.0163* | *-.069* | *-.004* |  |  |  |  |  |  |  |  |
| **PSQ-T** | **PSQ-W** | *-.025* | *.010* | *-.044* | *-.007* |  |  |  |  |  |  |  |  |

*Panel b: Independent variable: TQ; first intermediary variable:* ^Δ^*process variable_1_; second intermediary variable:* ^Δ^*process variable_2_; dependent variable:* ^Δ^*SES_aff.*

|  |  | | *a_1_* | *se* | *LLCI* | | *ULCI* | | *a_2_* | *se* | | *LLCI* | *ULCI* | | *a_3_* | | *se* | *LLCI* | | *ULCI* | *b_1_* | | *se* | | *LLCI* | *ULCI* | | *b_2_* | *se* | | *LLCI* | | *ULCI* |
| --- | --- | --- | --- | --- | --- | --- | --- | --- | --- | --- | --- | --- | --- | --- | --- | --- | --- | --- | --- | --- | --- | --- | --- | --- | --- | --- | --- | --- | --- | --- | --- | --- | --- |
| ^Δ^**ADS** | ^Δ^**ISR-D** | | *-.093* | *.016* | *-.124* | | *-.061* | |  |  | |  |  | | *.031* | | *.002* | *.027* | | *.036* | *.166* | | *.029* | | *.110* | *.223* | | *3.474* | *.379* | | *2.730* | | *4.218* |
| ^Δ^**ADS** | ^Δ^**PSQ-T** | | *-.096* | *.016* | *-.126* | | *-.065* | | *.001* | *.000* | | *.000* | *.001* | | *.006* | | *.001* | *.005* | | *.007* | *.232* | | *.028* | | *.178* | *.286* | | *9.158* | *1.610* | | *5.999* | | *12.318* |
| ^Δ^**ADS** | ^Δ^**PSQ-W** | | *-.096* | *.016* | *-.126* | | *-.065* | | *.000* | *.000* | | *.000* | *.001* | | *.005* | | *.001* | *.004* | | *.006* | *.229* | | *.027* | | *.176* | *.282* | | *11.487* | *1.653* | | *8.243* | | *14.730* |
| **Indirect effects** | | | *a_1_ b_1_* | | | *se* | | *LLCI* | | | *ULCI* | | | *a_2_ b_2_* | | *se* | | | *LLCI* | | | *ULCI* | | *a_1_ a_3_ b_2_* | | | *se* | | | *LLCI* | | *ULCI* | |
| ^Δ^**ADS** | | ^Δ^**ISR-D** | *-.015* | | | *.004* | | *-.024* | | | *-.008* | | |  | |  | | |  | | |  | | *-.010* | | | *.003* | | | *-.016* | | *-.005* | |
| ^Δ^**ADS** | | ^Δ^**PSQ-T** | *-.022* | | | *.005* | | *-.032* | | | *-.014* | | | *.005* | | *.003* | | | *.000* | | | *.010* | | *-.005* | | | *.002* | | | *-.008* | | *-.003* | |
| ^Δ^**ADS** | | ^Δ^**PSQ-W** | *-.022* | | | *.005* | | *-.032* | | | *-.014* | | |  | |  | | |  | | |  | | *-.005* | | | *.002* | | | *-.009* | | *-.003* | |

*Panel c: Independent variable:* ^Δ^*TQ; first intermediary variable:* ^Δ^*process variable_1_; second intermediary variable:* ^Δ^*process variable_2_; dependent variable:* ^Δ^*SES_aff.*

|  | |  | *a_1_* | *se* | | *LLCI* | *ULCI* | | *a_2_* | *se* | *LLCI* | | *ULCI* | *a_3_* | | *se* | | *LLCI* | *ULCI* | | *b_1_* | *se* | | *LLCI* | *ULCI* | *b_2_* | | *se* | *LLCI* | | *ULCI* | |
| --- | --- | --- | --- | --- | --- | --- | --- | --- | --- | --- | --- | --- | --- | --- | --- | --- | --- | --- | --- | --- | --- | --- | --- | --- | --- | --- | --- | --- | --- | --- | --- | --- |
| ^Δ^**ADS** | | ^Δ^**ISR-D** | *.339* | *.027* | | *.286* | *.392* | | *.015* | *.002* | *.011* | | *.019* | *.024* | | *.002* | | *.019* | *.029* | | *.136* | *.029* | | *.079* | *.193* | *3.053* | | *.384* | *2.299* | | *3.806* | |
| ^Δ^**ADS** | | ^Δ^**PSQ-T** | *.348* | *.026* | | *.296* | *.400* | | *.006* | *.001* | *.005* | | *.007* | *.003* | | *.001* | | *.002* | *.004* | | *.198* | *.028* | | *.143* | *.253* | *6.073* | | *1.708* | *2.722* | | *9.425* | |
| ^Δ^**ADS** | | ^Δ^**PSQ-W** | *.348* | *.026* | | *.296* | *.400* | | *.005* | *.001* | *.004* | | *.006* | *.003* | | *.001* | | *.002* | *.004* | | *.193* | *.028* | | *.139* | *.247* | *8.919* | | *1.718* | *5.547* | | *12.291* | |
| ^Δ^**ISR-D** | | ^Δ^**PSQ-T** | *.023* | *.002* | | *.019* | *.027* | | *.006* | *.001* | *.005* | | *.007* | *.047* | | *.007* | | *.033* | *.060* | | *3.240* | *.362* | | *2.529* | *3.951* | *4.003* | | *1.638* | *.788* | | *7.218* | |
| ^Δ^**ISR-D** | | ^Δ^**PSQ-W** | *.023* | *.002* | | *.019* | *.027* | | *.005* | *.000* | *.004* | | *.006* | *.045* | | *.007* | | *.032* | *.058* | | *3.098* | *.360* | | *2.391* | *3.805* | *7.321* | | *1.706* | *3.974* | | *10.668* | |
| ^Δ^**PSQ-T** | | ^Δ^**PSQ-W** | *.007* | *.000* | | *.006* | *.008* | | *.004* | *.000* | *.003* | | *.004* | *.326* | | *.028* | | *.271* | *.381* | | *4.699* | *1.692* | | *1.380* | *8.019* | *9.583* | | *1.759* | *6.132* | | *13.033* | |
| **Indirect effects** | | | *a_1_ b_1_* | | *se* | | | *LLCI* | | *ULCI* | | *a_2_ b_2_* | | | *se* | | *LLCI* | | | *ULCI* | | | *a_1_ a_3_ b_2_* | | *se* | | *LLCI* | | | *ULCI* | |  |
| ^Δ^**ADS** | ^Δ^**ISR-D** | | *.046* | | *.013* | | | *.021* | | *.073* | | *.045* | | | *.012* | | *.023* | | | *.071* | | | *.025* | | *.008* | | *.012* | | | *.041* | |  |
| ^Δ^**ADS** | ^Δ^**PSQ-T** | | *.069* | | *.013* | | | *.044* | | *.096* | | *.034* | | | *.011* | | *.013* | | | *.057* | | | *.007* | | *.003* | | *.002* | | | *.014* | |  |
| ^Δ^**ADS** | ^Δ^**PSQ-W** | | *.067* | | *.014* | | | *.043* | | *.096* | | *.041* | | | *.010* | | *.022* | | | *.062* | | | *.009* | | *.003* | | *.003* | | | *.016* | |  |
| ^Δ^**ISR-D** | ^Δ^**PSQ-T** | | *.074* | | *.018* | | | *.042* | | *.110* | | *.023* | | | *.010* | | *.004* | | | *.043* | | | *.004* | | *.002* | | *.001* | | | *.009* | |  |
| ^Δ^**ISR-D** | ^Δ^**PSQ-W** | | *.070* | | *.017* | | | *.039* | | *.107* | | *.034* | | | *.010* | | *.015* | | | *.055* | | | *.008* | | *.003* | | *.003* | | | *.014* | |  |
| ^Δ^**PSQ-T** | ^Δ^**PSQ-W** | | *.032* | | *.013* | | | *.008* | | *.058* | | *.034* | | | *.009* | | *.018* | | | *.052* | | | *.021* | | *.006* | | *.011* | | | *.033* | |  |

*Notes*: TQ = Tinnitus Questionnaire - German version; SES_aff = Affective Pain Perception Scale; ADS = Center for Epidemiological Studies Depression Scale (“Allgemeine Depressionsskala”); ISR-D = ICD-10 Symptom Rating total score - depressive syndrome; PSQ-T = Perceived Stress Questionnaire - tension; PSQ-W = Perceived Stress Questionnaire - worries; se = standard error; LLCL = lower level confidence interval; ULCL = upper level confidence interval.
